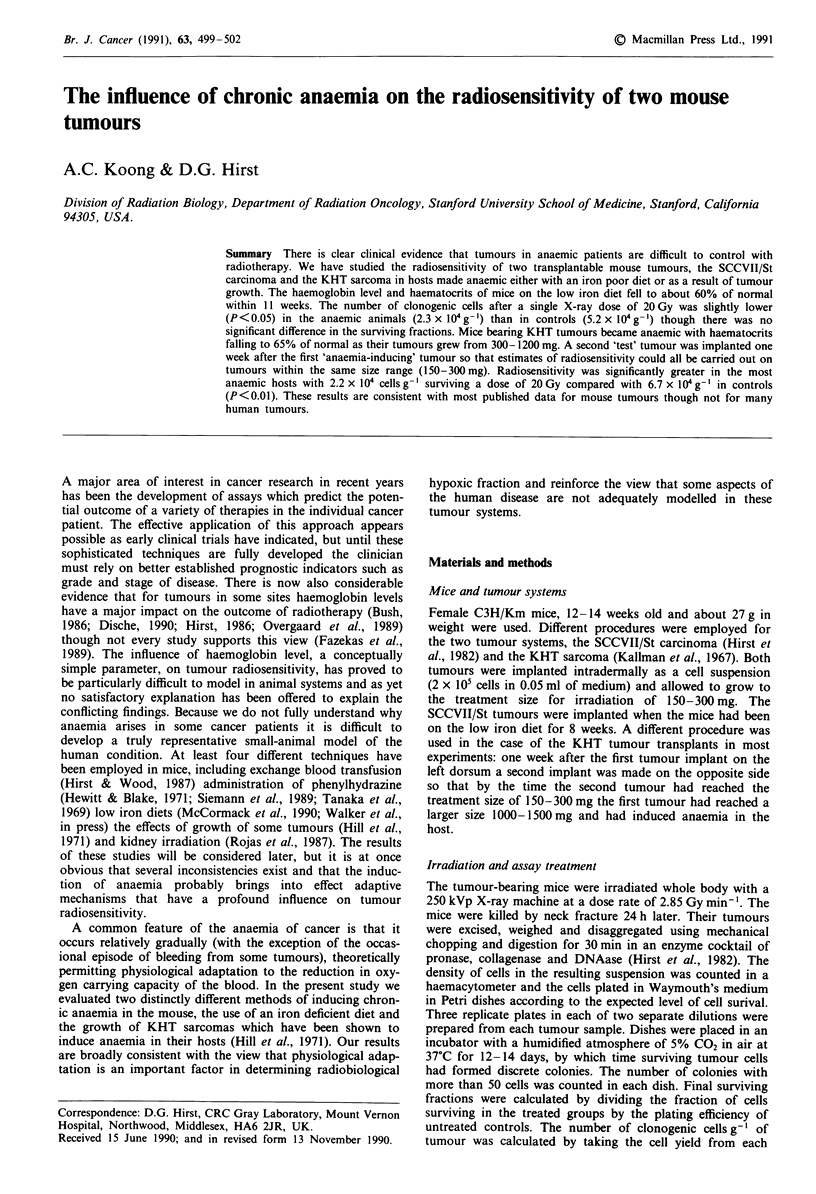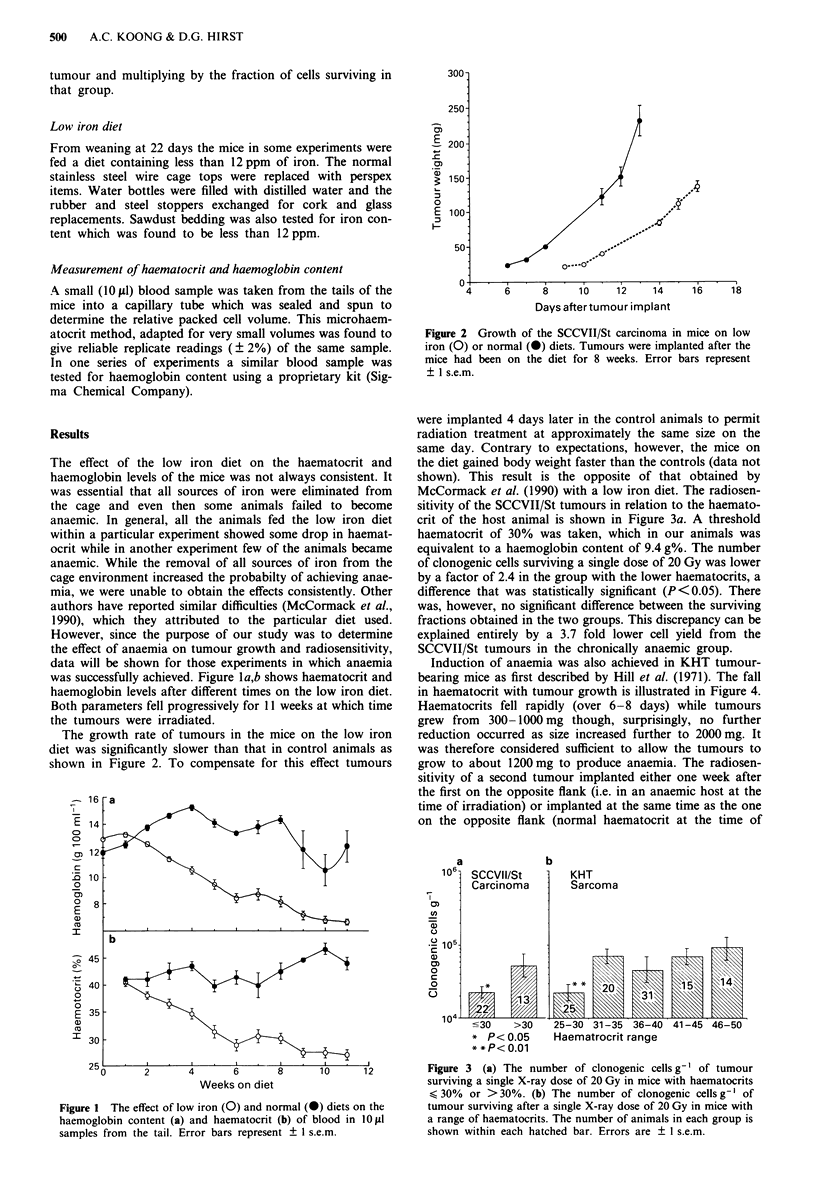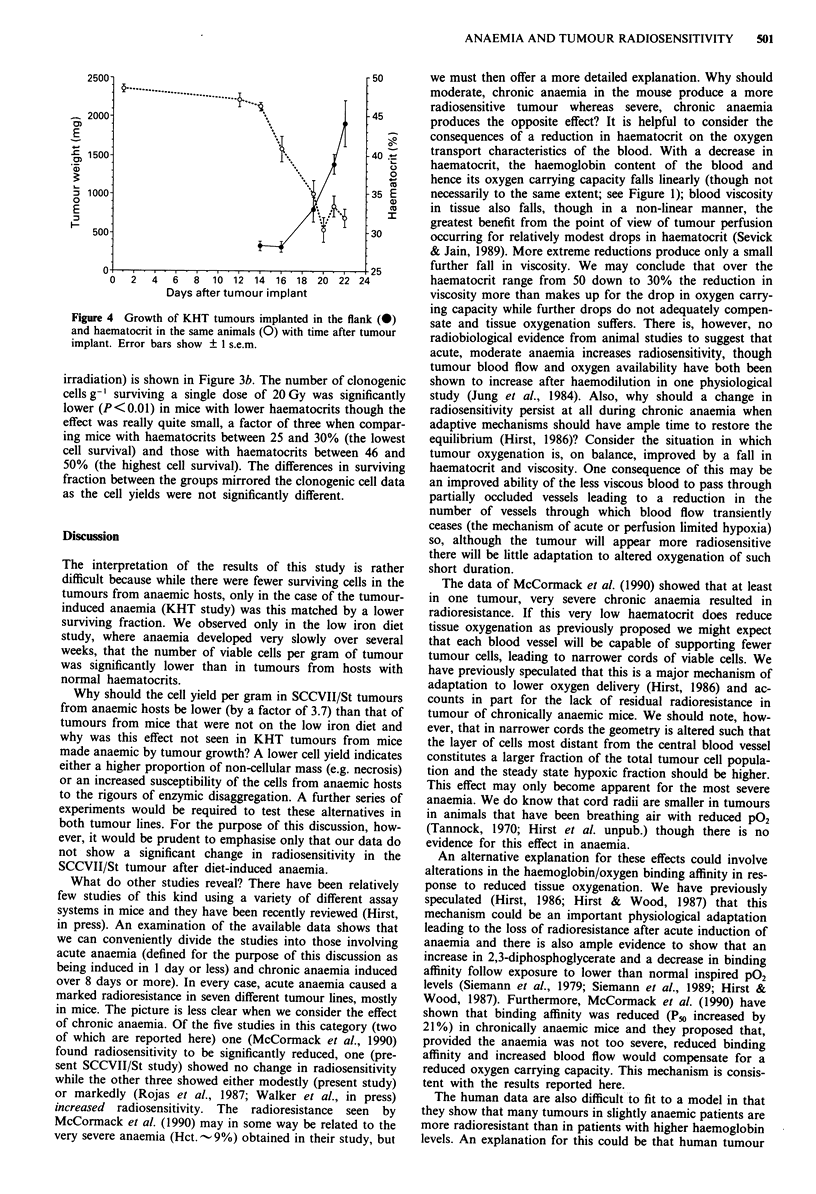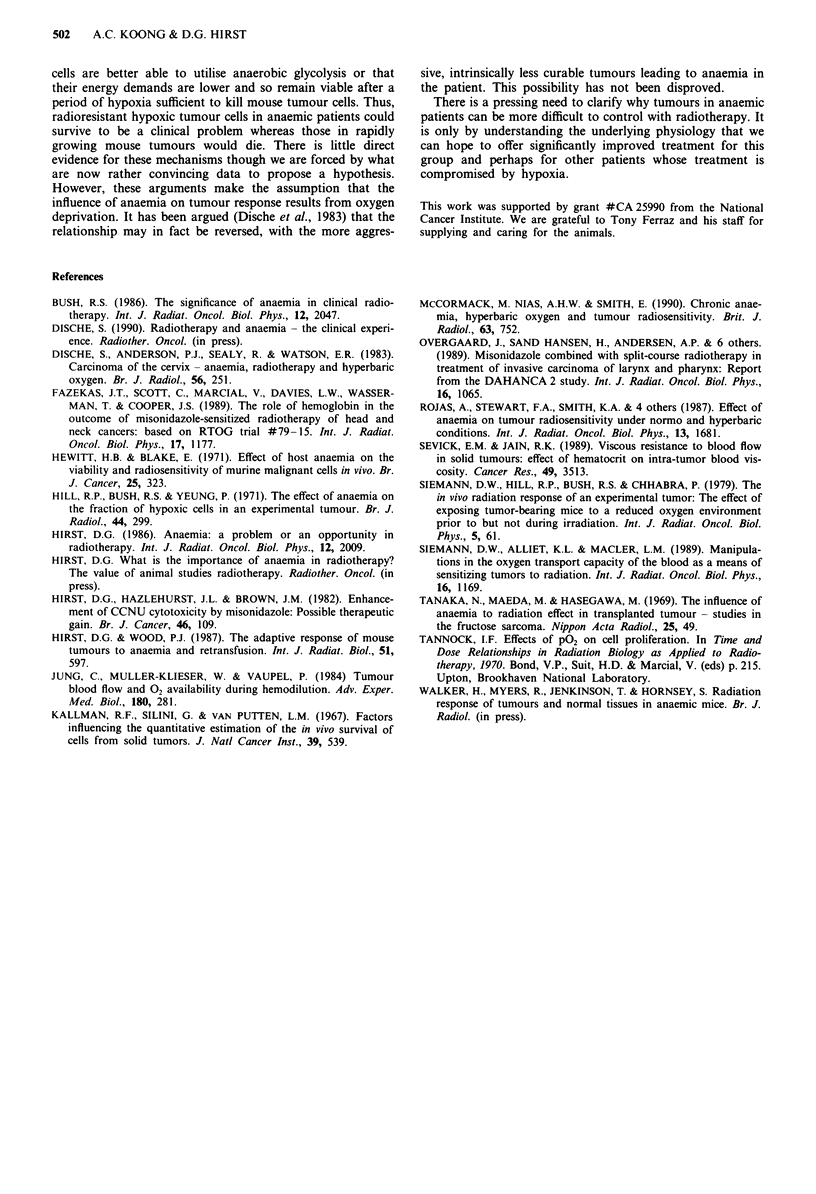# The influence of chronic anaemia on the radiosensitivity of two mouse tumours.

**DOI:** 10.1038/bjc.1991.119

**Published:** 1991-04

**Authors:** A. C. Koong, D. G. Hirst

**Affiliations:** Department of Radiation Oncology, Stanford University School of Medicine, California 94303.

## Abstract

There is clear clinical evidence that tumours in anaemic patients are difficult to control with radiotherapy. We have studied the radiosensitivity of two transplantable mouse tumours, the SCCVII/St carcinoma and the KHT sarcoma in hosts made anaemic either with an iron poor diet or as a result of tumour growth. The haemoglobin level and haematocrits of mice on the low iron diet fell to about 60% of normal within 11 weeks. The number of clonogenic cells after a single X-ray dose of 20 Gy was slightly lower (P less than 0.05) in the anaemic animals (2.3 X 10(4) g-1) than in controls (5.2 X 10(4) g-1) though there was no significant difference in the surviving fractions. Mice bearing KHT tumours became anaemic with haematocrits falling to 65% of normal as their tumours grew from 300-1200 mg. A second 'test' tumour was implanted one week after the first 'anaemia-inducing' tumour so that estimates of radiosensitivity could all be carried out on tumours within the same size range (150-300 mg). Radiosensitivity was significantly greater in the most anaemic hosts with 2.2 X 10(4) cells g-1 surviving a dose of 20 Gy compared with 6.7 x 10(4) g-1 in controls (P less than 0.01). These results are consistent with most published data for mouse tumours though not for many human tumours.


Br. J. Cancer (1991), 63, 499-502 © Macmillan Press Ltd., 1991

### The influence of chronic anaemia on the radiosensitivity of two mouse tumours

A.C. Koong & D.G. Hirst

*Division of Radiation Biology, Department of Radiation Oncology, Stanford University School of Medicine, Stanford, California 94305, USA.*

**Summary** There is clear clinical evidence that tumours in anaemic patients are difficult to control with radiotherapy. We have studied the radiosensitivity of two transplantable mouse tumours, the SCCVII/St carcinoma and the KHT sarcoma in hosts made anaemic either with an iron poor diet or as a result of tumour growth. The haemoglobin level and haematocrits of mice on the low iron diet fell to about 60% of normal within 11 weeks. The number of clonogenic cells after a single X-ray dose of 20 Gy was slightly lower ($P<0.05$) in the anaemic animals ($2.3 \times 10^4 g^{-1}$) than in controls ($5.2 \times 10^4 g^{-1}$) though there was no significant difference in the surviving fractions. Mice bearing KHT tumours became anaemic with haematocrits falling to 65% of normal as their tumours grew from 300-1200 mg. A second 'test' tumour was implanted one week after the first 'anaemia-inducing' tumour so that estimates of radiosensitivity could all be carried out on tumours within the same size range (150-300 mg). Radiosensitivity was significantly greater in the most anaemic hosts with $2.2 \times 10^4$ cells $g^{-1}$ surviving a dose of 20 Gy compared with $6.7 \times 10^4 g^{-1}$ in controls ($P<0.01$). These results are consistent with most published data for mouse tumours though not for many human tumours.

A major area of interest in cancer research in recent years has been the development of assays which predict the potential outcome of a variety of therapies in the individual cancer patient. The effective application of this approach appears possible as early clinical trials have indicated, but until these sophisticated techniques are fully developed the clinician must rely on better established prognostic indicators such as grade and stage of disease. There is now also considerable evidence that for tumours in some sites haemoglobin levels have a major impact on the outcome of radiotherapy (Bush, 1986; Dische, 1990; Hirst, 1986; Overgaard *et al.*, 1989) though not every study supports this view (Fazekas *et al.*, 1989). The influence of haemoglobin level, a conceptually simple parameter, on tumour radiosensitivity, has proved to be particularly difficult to model in animal systems and as yet no satisfactory explanation has been offered to explain the conflicting findings. Because we do not fully understand why anaemia arises in some cancer patients it is difficult to develop a truly representative small-animal model of the human condition. At least four different techniques have been employed in mice, including exchange blood transfusion (Hirst & Wood, 1987) administration of phenylhydrazine (Hewitt & Blake, 1971; Siemann *et al.*, 1989; Tanaka *et al.*, 1969) low iron diets (McCormack *et al.*, 1990; Walker *et al.*, in press) the effects of growth of some tumours (Hill *et al.*, 1971) and kidney irradiation (Rojas *et al.*, 1987). The results of these studies will be considered later, but it is at once obvious that several inconsistencies exist and that the induction of anaemia probably brings into effect adaptive mechanisms that have a profound influence on tumour radiosensitivity.

A common feature of the anaemia of cancer is that it occurs relatively gradually (with the exception of the occasional episode of bleeding from some tumours), theoretically permitting physiological adaptation to the reduction in oxygen carrying capacity of the blood. In the present study we evaluated two distinctly different methods of inducing chronic anaemia in the mouse, the use of an iron deficient diet and the growth of KHT sarcomas which have been shown to induce anaemia in their hosts (Hill *et al.*, 1971). Our results are broadly consistent with the view that physiological adaptation is an important factor in determining radiobiological hypoxic fraction and reinforce the view that some aspects of the human disease are not adequately modelled in these tumour systems.

Correspondence: D.G. Hirst, CRC Gray Laboratory, Mount Vernon Hospital, Northwood, Middlesex, HA6 2JR, UK.
Received 15 June 1990; and in revised form 13 November 1990.

### Materials and methods

### *Mice and tumour systems*

Female C3H/Km mice, 12-14 weeks old and about 27 g in weight were used. Different procedures were employed for the two tumour systems, the SCCVII/St carcinoma (Hirst *et al.*, 1982) and the KHT sarcoma (Kallman *et al.*, 1967). Both tumours were implanted intradermally as a cell suspension ($2 \times 10^5$ cells in 0.05 ml of medium) and allowed to grow to the treatment size for irradiation of 150-300 mg. The SCCVII/St tumours were implanted when the mice had been on the low iron diet for 8 weeks. A different procedure was used in the case of the KHT tumour transplants in most experiments: one week after the first tumour implant on the left dorsum a second implant was made on the opposite side so that by the time the second tumour had reached the treatment size of 150-300 mg the first tumour had reached a larger size 1000-1500 mg and had induced anaemia in the host.

### *Irradiation and assay treatment*

The tumour-bearing mice were irradiated whole body with a 250 kVp X-ray machine at a dose rate of 2.85 Gy $min^{-1}$. The mice were killed by neck fracture 24 h later. Their tumours were excised, weighed and disaggregated using mechanical chopping and digestion for 30 min in an enzyme cocktail of pronase, collagenase and DNAase (Hirst *et al.*, 1982). The density of cells in the resulting suspension was counted in a haemacytometer and the cells plated in Waymouth's medium in Petri dishes according to the expected level of cell surival. Three replicate plates in each of two separate dilutions were prepared from each tumour sample. Dishes were placed in an incubator with a humidified atmosphere of 5% $CO_2$ in air at 37°C for 12-14 days, by which time surviving tumour cells had formed discrete colonies. The number of colonies with more than 50 cells was counted in each dish. Final surviving fractions were calculated by dividing the fraction of cells surviving in the treated groups by the plating efficiency of untreated controls. The number of clonogenic cells $g^{-1}$ of tumour was calculated by taking the cell yield from each tumour and multiplying by the fraction of cells surviving in that group.

*Low iron diet*

From weaning at 22 days the mice in some experiments were fed a diet containing less than 12 ppm of iron. The normal stainless steel wire cage tops were replaced with perspex items. Water bottles were filled with distilled water and the rubber and steel stoppers exchanged for cork and glass replacements. Sawdust bedding was also tested for iron content which was found to be less than 12 ppm.

*Measurement of haematocrit and haemoglobin content*

A small (10 µl) blood sample was taken from the tails of the mice into a capillary tube which was sealed and spun to determine the relative packed cell volume. This microhaematocrit method, adapted for very small volumes was found to give reliable replicate readings (± 2%) of the same sample. In one series of experiments a similar blood sample was tested for haemoglobin content using a proprietary kit (Sigma Chemical Company).

### Results

The effect of the low iron diet on the haematocrit and haemoglobin levels of the mice was not always consistent. It was essential that all sources of iron were eliminated from the cage and even then some animals failed to become anaemic. In general, all the animals fed the low iron diet within a particular experiment showed some drop in haematocrit while in another experiment few of the animals became anaemic. While the removal of all sources of iron from the cage environment increased the probabilty of achieving anaemia, we were unable to obtain the effects consistently. Other authors have reported similar difficulties (McCormack *et al.*, 1990), which they attributed to the particular diet used. However, since the purpose of our study was to determine the effect of anaemia on tumour growth and radiosensitivity, data will be shown for those experiments in which anaemia was successfully achieved. Figure 1*a*,*b* shows haematocrit and haemoglobin levels after different times on the low iron diet. Both parameters fell progressively for 11 weeks at which time the tumours were irradiated.

The growth rate of tumours in the mice on the low iron diet was significantly slower than that in control animals as shown in Figure 2. To compensate for this effect tumours

**Figure 1** The effect of low iron (O) and normal (●) diets on the haemoglobin content (**a**) and haematocrit (**b**) of blood in 10 µl samples from the tail. Error bars represent ± 1 s.e.m.

**Figure 2** Growth of the SCCVII/St carcinoma in mice on low iron (O) or normal (●) diets. Tumours were implanted after the mice had been on the diet for 8 weeks. Error bars represent ± 1 s.e.m.

were implanted 4 days later in the control animals to permit radiation treatment at approximately the same size on the same day. Contrary to expectations, however, the mice on the diet gained body weight faster than the controls (data not shown). This result is the opposite of that obtained by McCormack *et al.* (1990) with a low iron diet. The radiosensitivity of the SCCVII/St tumours in relation to the haematocrit of the host animal is shown in Figure 3*a*. A threshold haematocrit of 30% was taken, which in our animals was equivalent to a haemoglobin content of 9.4 g%. The number of clonogenic cells surviving a single dose of 20 Gy was lower by a factor of 2.4 in the group with the lower haematocrits, a difference that was statistically significant ($P < 0.05$). There was, however, no significant difference between the surviving fractions obtained in the two groups. This discrepancy can be explained entirely by a 3.7 fold lower cell yield from the SCCVII/St tumours in the chronically anaemic group.

Induction of anaemia was also achieved in KHT tumour-bearing mice as first described by Hill *et al.* (1971). The fall in haematocrit with tumour growth is illustrated in Figure 4. Haematocrits fell rapidly (over 6–8 days) while tumours grew from 300–1000 mg though, surprisingly, no further reduction occurred as size increased further to 2000 mg. It was therefore considered sufficient to allow the tumours to grow to about 1200 mg to produce anaemia. The radiosensitivity of a second tumour implanted either one week after the first on the opposite flank (i.e. in an anaemic host at the time of irradiation) or implanted at the same time as the one on the opposite flank (normal haematocrit at the time of

**Figure 3** (**a**) The number of clonogenic cells $g^{-1}$ of tumour surviving a single X-ray dose of 20 Gy in mice with haematocrits ≤ 30% or > 30%. (**b**) The number of clonogenic cells $g^{-1}$ of tumour surviving after a single X-ray dose of 20 Gy in mice with a range of haematocrits. The number of animals in each group is shown within each hatched bar. Errors are ± 1 s.e.m.

**Figure 4** Growth of KHT tumours implanted in the flank (●) and haematocrit in the same animals (○) with time after tumour implant. Error bars show ± 1 s.e.m.

irradiation) is shown in Figure 3*b*. The number of clonogenic cells $g^{-1}$ surviving a single dose of 20 Gy was significantly lower ($P < 0.01$) in mice with lower haematocrits though the effect was really quite small, a factor of three when comparing mice with haematocrits between 25 and 30% (the lowest cell survival) and those with haematocrits between 46 and 50% (the highest cell survival). The differences in surviving fraction between the groups mirrored the clonogenic cell data as the cell yields were not significantly different.

### Discussion

The interpretation of the results of this study is rather difficult because while there were fewer surviving cells in the tumours from anaemic hosts, only in the case of the tumour-induced anaemia (KHT study) was this matched by a lower surviving fraction. We observed only in the low iron diet study, where anaemia developed very slowly over several weeks, that the number of viable cells per gram of tumour was significantly lower than in tumours from hosts with normal haematocrits.

Why should the cell yield per gram in SCCVII/St tumours from anaemic hosts be lower (by a factor of 3.7) than that of tumours from mice that were not on the low iron diet and why was this effect not seen in KHT tumours from mice made anaemic by tumour growth? A lower cell yield indicates either a higher proportion of non-cellular mass (e.g. necrosis) or an increased susceptibility of the cells from anaemic hosts to the rigours of enzymic disaggregation. A further series of experiments would be required to test these alternatives in both tumour lines. For the purpose of this discussion, however, it would be prudent to emphasise only that our data do not show a significant change in radiosensitivity in the SCCVII/St tumour after diet-induced anaemia.

What do other studies reveal? There have been relatively few studies of this kind using a variety of different assay systems in mice and they have been recently reviewed (Hirst, in press). An examination of the available data shows that we can conveniently divide the studies into those involving acute anaemia (defined for the purpose of this discussion as being induced in 1 day or less) and chronic anaemia induced over 8 days or more). In every case, acute anaemia caused a marked radioresistance in seven different tumour lines, mostly in mice. The picture is less clear when we consider the effect of chronic anaemia. Of the five studies in this category (two of which are reported here) one (McCormack *et al.*, 1990) found radiosensitivity to be significantly reduced, one (present SCCVII/St study) showed no change in radiosensitivity while the other three showed either modestly (present study) or markedly (Rojas *et al.*, 1987; Walker *et al.*, in press) *increased* radiosensitivity. The radioresistance seen by McCormack *et al.* (1990) may in some way be related to the very severe anaemia (Hct. ~9%) obtained in their study, but we must then offer a more detailed explanation. Why should moderate, chronic anaemia in the mouse produce a more radiosensitive tumour whereas severe, chronic anaemia produces the opposite effect? It is helpful to consider the consequences of a reduction in haematocrit on the oxygen transport characteristics of the blood. With a decrease in haematocrit, the haemoglobin content of the blood and hence its oxygen carrying capacity falls linearly (though not necessarily to the same extent; see Figure 1); blood viscosity in tissue also falls, though in a non-linear manner, the greatest benefit from the point of view of tumour perfusion occurring for relatively modest drops in haematocrit (Sevick & Jain, 1989). More extreme reductions produce only a small further fall in viscosity. We may conclude that over the haematocrit range from 50 down to 30% the reduction in viscosity more than makes up for the drop in oxygen carrying capacity while further drops do not adequately compensate and tissue oxygenation suffers. There is, however, no radiobiological evidence from animal studies to suggest that acute, moderate anaemia increases radiosensitivity, though tumour blood flow and oxygen availability have both been shown to increase after haemodilution in one physiological study (Jung *et al.*, 1984). Also, why should a change in radiosensitivity persist at all during chronic anaemia when adaptive mechanisms should have ample time to restore the equilibrium (Hirst, 1986)? Consider the situation in which tumour oxygenation is, on balance, improved by a fall in haematocrit and viscosity. One consequence of this may be an improved ability of the less viscous blood to pass through partially occluded vessels leading to a reduction in the number of vessels through which blood flow transiently ceases (the mechanism of acute or perfusion limited hypoxia) so, although the tumour will appear more radiosensitive there will be little adaptation to altered oxygenation of such short duration.

The data of McCormack *et al.* (1990) showed that at least in one tumour, very severe chronic anaemia resulted in radioresistance. If this very low haematocrit does reduce tissue oxygenation as previously proposed we might expect that each blood vessel will be capable of supporting fewer tumour cells, leading to narrower cords of viable cells. We have previously speculated that this is a major mechanism of adaptation to lower oxygen delivery (Hirst, 1986) and accounts in part for the lack of residual radioresistance in tumour of chronically anaemic mice. We should note, however, that in narrower cords the geometry is altered such that the layer of cells most distant from the central blood vessel constitutes a larger fraction of the total tumour cell population and the steady state hypoxic fraction should be higher. This effect may only become apparent for the most severe anaemia. We do know that cord radii are smaller in tumours in animals that have been breathing air with reduced $pO_2$ (Tannock, 1970; Hirst *et al.* unpub.) though there is no evidence for this effect in anaemia.

An alternative explanation for these effects could involve alterations in the haemoglobin/oxygen binding affinity in response to reduced tissue oxygenation. We have previously speculated (Hirst, 1986; Hirst & Wood, 1987) that this mechanism could be an important physiological adaptation leading to the loss of radioresistance after acute induction of anaemia and there is also ample evidence to show that an increase in 2,3-diphosphoglycerate and a decrease in binding affinity follow exposure to lower than normal inspired $pO_2$ levels (Siemann *et al.*, 1979; Siemann *et al.*, 1989; Hirst & Wood, 1987). Furthermore, McCormack *et al.* (1990) have shown that binding affinity was reduced ($P_{50}$ increased by 21%) in chronically anaemic mice and they proposed that, provided the anaemia was not too severe, reduced binding affinity and increased blood flow would compensate for a reduced oxygen carrying capacity. This mechanism is consistent with the results reported here.

The human data are also difficult to fit to a model in that they show that many tumours in slightly anaemic patients are more radioresistant than in patients with higher haemoglobin levels. An explanation for this could be that human tumour cells are better able to utilise anaerobic glycolysis or that their energy demands are lower and so remain viable after a period of hypoxia sufficient to kill mouse tumour cells. Thus, radioresistant hypoxic tumour cells in anaemic patients could survive to be a clinical problem whereas those in rapidly growing mouse tumours would die. There is little direct evidence for these mechanisms though we are forced by what are now rather convincing data to propose a hypothesis. However, these arguments make the assumption that the influence of anaemia on tumour response results from oxygen deprivation. It has been argued (Dische *et al.*, 1983) that the relationship may in fact be reversed, with the more aggressive, intrinsically less curable tumours leading to anaemia in the patient. This possibility has not been disproved.

There is a pressing need to clarify why tumours in anaemic patients can be more difficult to control with radiotherapy. It is only by understanding the underlying physiology that we can hope to offer significantly improved treatment for this group and perhaps for other patients whose treatment is compromised by hypoxia.

This work was supported by grant #CA 25990 from the National Cancer Institute. We are grateful to Tony Ferraz and his staff for supplying and caring for the animals.

### References

BUSH, R.S. (1986). The significance of anaemia in clinical radiotherapy. *Int. J. Radiat. Oncol. Biol. Phys.*, **12**, 2047.

DISCHE, S. (1990). Radiotherapy and anaemia – the clinical experience. *Radiother. Oncol.* (in press).

DISCHE, S., ANDERSON, P.J., SEALY, R. & WATSON, E.R. (1983). Carcinoma of the cervix – anaemia, radiotherapy and hyperbaric oxygen. *Br. J. Radiol.*, **56**, 251.

FAZEKAS, J.T., SCOTT, C., MARCIAL, V., DAVIES, L.W., WASSERMAN, T. & COOPER, J.S. (1989). The role of hemoglobin in the outcome of misonidazole-sensitized radiotherapy of head and neck cancers: based on RTOG trial #79-15. *Int. J. Radiat. Oncol. Biol. Phys.*, **17**, 1177.

HEWITT, H.B. & BLAKE, E. (1971). Effect of host anaemia on the viability and radiosensitivity of murine malignant cells *in vivo*. *Br. J. Cancer*, **25**, 323.

HILL, R.P., BUSH, R.S. & YEUNG, P. (1971). The effect of anaemia on the fraction of hypoxic cells in an experimental tumour. *Br. J. Radiol.*, **44**, 299.

HIRST, D.G. (1986). Anaemia: a problem or an opportunity in radiotherapy. *Int. J. Radiat. Oncol. Biol. Phys.*, **12**, 2009.

HIRST, D.G. What is the importance of anaemia in radiotherapy? The value of animal studies radiotherapy. *Radiother. Oncol.* (in press).

HIRST, D.G., HAZLEHURST, J.L. & BROWN, J.M. (1982). Enhancement of CCNU cytotoxicity by misonidazole: Possible therapeutic gain. *Br. J. Cancer*, **46**, 109.

HIRST, D.G. & WOOD, P.J. (1987). The adaptive response of mouse tumours to anaemia and retransfusion. *Int. J. Radiat. Biol.*, **51**, 597.

JUNG, C., MULLER-KLIESER, W. & VAUPEL, P. (1984) Tumour blood flow and $O_2$ availability during hemodilution. *Adv. Exper. Med. Biol.*, **180**, 281.

KALLMAN, R.F., SILINI, G. & VAN PUTTEN, L.M. (1967). Factors influencing the quantitative estimation of the *in vivo* survival of cells from solid tumors. *J. Natl Cancer Inst.*, **39**, 539.

MCCORMACK, M. NIAS, A.H.W. & SMITH, E. (1990). Chronic anaemia, hyperbaric oxygen and tumour radiosensitivity. *Brit. J. Radiol.*, **63**, 752.

OVERGAARD, J., SAND HANSEN, H., ANDERSEN, A.P. & 6 others. (1989). Misonidazole combined with split-course radiotherapy in treatment of invasive carcinoma of larynx and pharynx: Report from the DAHANCA 2 study. *Int. J. Radiat. Oncol. Biol. Phys.*, **16**, 1065.

ROJAS, A., STEWART, F.A., SMITH, K.A. & 4 others (1987). Effect of anaemia on tumour radiosensitivity under normo and hyperbaric conditions. *Int. J. Radiat. Oncol. Biol. Phys.*, **13**, 1681.

SEVICK, E.M. & JAIN, R.K. (1989). Viscous resistance to blood flow in solid tumours: effect of hematocrit on intra-tumor blood viscosity. *Cancer Res.*, **49**, 3513.

SIEMANN, D.W., HILL, R.P., BUSH, R.S. & CHHABRA, P. (1979). The *in vivo* radiation response of an experimental tumor: The effect of exposing tumor-bearing mice to a reduced oxygen environment prior to but not during irradiation. *Int. J. Radiat. Oncol. Biol. Phys.*, **5**, 61.

SIEMANN, D.W., ALLIET, K.L. & MACLER, L.M. (1989). Manipulations in the oxygen transport capacity of the blood as a means of sensitizing tumors to radiation. *Int. J. Radiat. Oncol. Biol. Phys.*, **16**, 1169.

TANAKA, N., MAEDA, M. & HASEGAWA, M. (1969). The influence of anaemia to radiation effect in transplanted tumour – studies in the fructose sarcoma. *Nippon Acta Radiol.*, **25**, 49.

TANNOCK, I.F. Effects of $pO_2$ on cell proliferation. In *Time and Dose Relationships in Radiation Biology as Applied to Radiotherapy, 1970*. Bond, V.P., Suit, H.D. & Marcial, V. (eds) p. 215. Upton, Brookhaven National Laboratory.

WALKER, H., MYERS, R., JENKINSON, T. & HORNSEY, S. Radiation response of tumours and normal tissues in anaemic mice. *Br. J. Radiol.* (in press).